# Baseline urinary ALA and PBG as criteria for starting pharmacologic prophylactic treatment in acute intermittent porphyria treated with givosiran

**DOI:** 10.1016/j.ymgmr.2024.101169

**Published:** 2024-12-05

**Authors:** Hung-Chou Kuo, Long-Sun Ro, Chia-Ni Lin, Chun-Che Chu, Ming-Feng Liao, Hong-Shiu Chang

**Affiliations:** a Department of Neurology, Chang Gung Memorial Hospital Linkou Medical Center and College of Medicine, Chang Gung University, Taoyuan, Taiwan; b Department of Laboratory Medicine, Chang Gung Memorial Hospital, Taoyuan, Taiwan; c Department of Medical Biotechnology and Laboratory Science, College of Medicine, Chang Gung University, Taoyuan, Taiwan

**Keywords:** Acute intermittent porphyria, delta-aminolevulinic acid, Porphobilinogen, Givosiran, diagnosis

## Abstract

**Introduction:**

For patients with acute intermittent porphyria (AIP), a true attack could be difficult to distinguish from chronic abdominal pain. This study focused on treatment responses from two patients with confirmed elevated biochemical data (delta-aminolevulinic acid (ALA), porphobilinogen (PBG)) and clinical evidence for acute attacks before starting givosiran.

**Methods:**

Data from patients who participated in the phase III givosiran trial in Taiwan between May 2018 and May 2021 were reviewed. The pre-trial and post-trial biochemical data (urinary ALA/PBG), annualized attack rate (AAR), for two participants were obtained from our hospital record.

**Results:**

Two patients had detailed records of biochemical evidence of acute attacks pre-trial (ALA:11.66–79.8 mg/24-h urine collection, PBG:75.45–160.11 mg/24-h). Patient Pb/Gn#1 with a disease duration of 1.6-years, had zero AAR during givosiran treatment. Patient Pb/Gn#2 had received prior hemin prophylaxis, had AIP for 6.7-years, had an AAR of 17.0 before givosiran, and an AAR of 12 at the post-trial compassionate-use period. The change in SF-12 PCS score from baseline was marginally clinical-meaningful (2.8 for Patient Pb/Gn#1 and 2.0 for Patient Pb/Gn#2).

**Conclusion:**

Our data from 2 AIP patients with biochemical and clinical evidence of acute attacks suggested that patient with a shorter disease duration may respond better in terms of AAR. Further studies are necessary to understand the association between disease characteristics, treatment history, and optimal treatment response for patients with recurrent AIP in terms of both attack frequency and quality of life.

## 1 Introduction

Acute porphyria (AP) comprises a group of four genetic disorders caused by mutations in enzymes that catalyze heme biosynthesis. [1] Regardless of the AP subtype, the resultant outcome is up-regulation of hepatic delta-aminolevulinic acid synthase 1 (ALAS1), which leads to the accumulation of the neurotoxic heme precursors delta-aminolevulinic acid (ALA) and porphobilinogen (PBG) and can cause potentially life-threatening acute attacks. [2,3] Symptoms during these attacks include severe abdominal pain, vomiting, tachycardia, and hypertension; severe attacks may be complicated by acute encephalopathy (seizures, hyponatremia, psychiatric symptoms), or acute polyneuropathy (paralysis, myalgia, etc.). [4] Long-term complications of recurrent attacks include chronic symptoms of autonomic neuropathy, encephalopathy and polyneuropathy, chronic kidney disease, liver cancer, and hypertension. [5] Recent studies have recommended the provision of pharmacologic prophylaxis for AIP patients experiencing ≥4 acute attacks per year, [6,7] however, there has been no consensus on the definition of an ‘acute attack’. [8] because chronic symptoms of abdominal pain are common in patients with recurrent AIP, making it difficult to distinguish from a true acute attack. [9]

Prophylactic therapy with hemin has been used in clinical settings for patients experiencing recurrent attacks. [[6], [7],^10^] Hemin prophylaxis, while effective, carries potential AE risks such as chronic iron overload and complications associated with intravenous access. [1,12] Additionally, there is also evidence suggesting that repeated hemin infusions may contribute to chronic inflammatory hepatic disease by inducing heme oxygenase-1 and perpetuating a high ALAS1 level, potentially exacerbating the recurrence of neurovisceral crises. [13] Given these concerns, recent clinical trials have explored alternative therapies such as givosiran, an small interfering RNA (siRNA) therapeutic that targets ALAS1 expression in the liver. [2,14]

Givosiran is a small interfering RNA (siRNA) approved by the US Federal Drug Administration in 2019 for treating patients with acute porphyria. It is designed to target liver cells, effectively inhibiting the expression of ALAS1 and thereby reducing the production and accumulation of the neurotoxic heme precursors ALA and PBG. The efficacy of givosiran in patients with recurrent acute attacks was investigated in phase III trials, [2,5] and real-world studies. [15] The final analysis of the 36-month ENVISION trial showed a sustained improvement in acute porphyria symptoms, an increase in the proportion of patients with no attacks or zero days of hemin use over time, and improved quality of life. [14,16] A multicenter study focusing on the real-world data of severe AIP patients treated with givosiran, observed that the individual responses to treatment were influenced by the duration of the disease course and the ALA levels. [15] This highlighted the importance of understanding individual ALA and PBG levels.

Given the variation in disease severity and baseline biochemical precursors of the patients included in the phase 3 trial [8], the difficulty in defining a true acute attack, as well as the limited studies reporting East-Asian regional-specific data, therefore, this study focused on the response to givosiran treatment from two AIP patients whose baseline ALA/PBG were monitored and acute attacks was confirmed clinically and biochemically before the givosiran trial.

## 2 Patients and methods

### 2.1 Patients

A total of seven patients were enrolled in the givosiran trial between May 2018 and May 2021 in Taiwan. [2,16]

All data collected during givosiran treatment period were obtained from the trial report, including baseline demographics, composite AAR, urinary ALA, PBG, annualized rate of administered hemin doses (annualized days of hemin use), and the patient's quality of life (Physical Component Summary (PCS) of the 12-item Short-form Health Survey (SF-12 PCS). The definition of composite AAR used in the trial was composed of attacks requiring hospitalization, an urgent healthcare visit, or intravenous hemin at home.

Regular monitoring of urinary ALA and PBG before the givosiran trial and during the post-trial compassionate-use program period for two of the patients were collected as part of the routine care in our hospital (Pb/Gn #1 and Pb/Gn #2). These two patients represented AIP patients whose acute attacks were confirmed with clinical and biochemical evidence in this study. Acute attack as defined in our hospital was an attack requiring a visit to the emergency department, or hospitalization, and were given heme arginate treatment.

### 2.2 Ethics

This study was conducted in accordance with the ethical principles outlined in the Helsinki Declaration and approval was obtained from the ethics committee of our Hospital (IRB number: 201900074B0, 202000914B0, and 201800347A4).

### 2.3 Statistical analysis

Continuous variables are presented as the median and (minimum, maximum); categorical variables are presented as counts and percentages. Demographic and clinical characteristics of the study population are summarized for each participant. All data tabulation was performed using Microsoft Excel (Microsoft, Redmond, WA, USA) and GraphPad Prism 6 (San Diego, CA, USA).

## 3 Results

The demographics and baseline characteristics of the two patients (Pb/Gn #1 and Pb/Gb#2) whose acute attacks were confirmed with clinical and biochemical evidence were presented in Table 1. Pb/Gn #1 has no prior prophylactic treatment, while Pb/Gn #2 patient was receiving weekly prophylactic heme arginate infusion until the washout period. The biochemical evidence of elevated urinary ALA and PBG indicating true acute attacks for the pre-trial and post-trial compassionate-use periods was presented in Table 2. ALA/PBG levels collected include close to attack onset, during heme treatment infusion, and close to symptom relief or remission. We observed that during compassionate givosiran treatment, the PBG/ALA levels at close to remission were lower compared to pre-trial levels for remission (Patient Pb/Gn#1), and the PBG/ALA levels during acute attacks were lower compared to pre-trial levels for attack (Patient Pb/Gn#2). Patient Pb/Gn#2 urinary ALA and PBG for an acute attack in compassionate-givosiran treatment period (ALA: 6.41–7.45 mg/24 h; PBG: 32.52–34.16 mg/24 h) were lower compared to the level for acute attacks before the trial (ALA: 11.66–79.8 mg/24 h; PBG: 75.45–160.11 mg/24 h) (Table 2).

**Table 1:** Demographic and disease characteristics of the two patients with monitoring of delta-aminolevulinic acid (ALA), and porphobilinogen (PBG), were used as biochemical evidence to confirm acute attacks, before the trial.

	Pb/Gn # 1	Pb/Gn # 2
Age, years	25–29	20–24
Sex	F	F
BMI, kg/m^2^	24.4	26.7
Weight, kg	59.3	65
Years of AIP before trial	1.6	6.7
HMBS gene mutation	c.517C > T	c.652G > A
PBGD activity, nmol/h/ml RBC⁎	19.5	18.3
Prior hemin prophylaxis regimen	No	Yes
Prior chronic symptoms when not having attacks	No	No
Baseline Urinary ALA (mmol/mol Cr)	17.31	26.72
Baseline Urinary PBG (mmol/mol Cr)	57.61	72.53

Data was presented as median or count.

Pb/Gn refers to the placebo (Pb) crossover arm of the ENVISION trial: patients received 6 months placebo during the double-blind period and had givosiran (Gn) through open-label extension.

AIP, acute intermittent porphyria, ALA, aminolevulinic acid; BMI, body mass index; Cr, creatinine; HMBS, hydroxymethylbilane Synthase PBG, porphobilinogen; PBGD, porphobilinogen deaminase.

⁎ References: PBGD activity 30.2–73.3 nmol/h/ml RBC.

**Table 2:** 24-h urinary of PBG and ALA (mg/24 h) for Pb/Gn #1 and Pb/Gn #2 before and after the phase 3 givosiran trial.

		Before phase 3 givosiran trial										Trial	post-trial compassionate givosiran treatment			
Pb/Gn #1	Date of collection	Nov.282016	Jan.22017	Mar.32017	May.42017	Jul.42017	Oct.82017	Nov.72017	Apr.112018			May. 2018-Apr. 2021	Oct.82021	^**+**^Dec.42021		
	Stage of attack	A	A	H	A	A	R	H	H				R	R		
	PBG	134.1	82.34	84.53	96.08	159.4	23.82	76.19	76.19				4.81	7.88		
	δ-ALA	74.85	46.64	10	55.59	69.76	4.4	12.29	12.29				4.17	3.57		
Pb/Gn #2	Date of collection	Nov.252011	Dec.62011	Jan.62012	Nov.232012	Dec.112012	Jan.172013	Dec.62013	Jan.242014	⁎Jan.292014	May.132018	Aug. 2018-May 2021	Jul.162021	Sep.242021	Nov.52021	Nov.122021
	Stage of attack	A	A	A	A	A	A	A	H	R	A		R	A	A	A
	PBG	101.73	154.26	160.11	125	143.75	125.8	75.45	39.67	26.1	122.89		13.44	N/A	32.52	34.16
	δ-ALA	33.1	55.73	65.73	49.31	79.8	39.31	11.66	8.52	7.67	41.79		3.3	6.64	7.45	6.41

A, H, R indicates the stage when the measurements were collected: A = close to attack onset and initial heme arginate infusion; H = during continued heme arginate infusion for acute attack; R = close to symptom relief or remission.

Pb/Gn, placebo (Pb) crossover arm receiving placebo during the 6-month double-blind (DB) period.

**+** Pb/Gn #1 stopped receiving compassionate givosiran in December 2021 due to a confirmed pregnancy in November. PBA/ALA levels were checked in December and at the same time, the pharmaceutical company was notified. The patient was recommended to discontinue givosiran treatment.

Date, Month-day-year; ALA, aminolevulinic acid; PBG, porphobilinogen.

Reference for PBG/24-h urine collection: 0.0–2.7 mg/24 h

Reference for δ-ALA/24-h urine collection: 1.5–7.5 mg/24 h

⁎ Heme arginate prophylactic treatment started.

The treatment outcomes defined by AAR and human hemin usage are summarized in Table 3. Patient Pb/Gn #1 did not receive prior prophylactic treatment and had an AAR of 8.7 before the trial. The composite AAR for Patient Pb/Gn #1 was zero during givosiran treatment. The historical AAR for patient Pb/Gn #2 before receiving weekly prophylactic treatment was 17.0, and she continued to experience higher composite AAR (7.4) and hemin-use (10.0) during givosiran treatment. However, the AAR during post-trial compassionate use (12.0) was lower than the AAR before givosiran treatment (17.0) (Table 3**).**

**Table 3:** Summary of AAR and human hemin usage for the two patients with biochemically confirmed disease severity at givosiran treatment initiation.

Time Period	Outcome	Pb/Gn #1	Pb/Gn #2
Before trial^b^	AAR	8.7	17.0^c^
	Human hemin, annualized day	14.7	30.0^c^
6-month placebo^a^	Composite AAR	4.3	25.9
	Human hemin, annualized day	2.1	45.4
During givosiran treatment (through 36 months)^a^	Composite AAR	1.7	7.4
	Human hemin, annualized day	0.0	10.0
Post-trial compassionate givosiran^b^	AAR	0.0	12.0
	Human hemin, annualized day	0.0	12.0

AAR, annualized attack rate. Acute attack as defined in our medical center was an attack requiring a visit to the emergency department, or hospitalization, and given heme arginate treatment.

Pb/Gn refers to the placebo (Pb) crossover arm of the ENVISION trial: patients received 6 months placebo in the double-blind period and received givosiran (Gn) in the open-label extension.

a The data for 6-month placebo and during givosiran treatment, including composite AAR and use of human hemin were reported by the phase 3 ENVISION trial. Composite AA as defined by ENVISION comprised of attacks requiring hospitalization, an urgent healthcare visit, or intravenous hemin at home.

b Before trial and post-trial data were collected from hospital medical records.

c Pb/Gn #2 patient was receiving weekly prophylactic heme arginate infusion until the washout period.

The AAR during the compassionate -treatment period for both Pb/Gn #1 and #2 was lower compared to the AAR before the trial and during the 6-month placebo period, indicating improvement in clinical presentation. Patient Pb/Gn #1 had zero AAR during post-trial compassionate givosiran treatment, while patient Pb/Gn #2 continued to experience acute attacks (AAR: 12) during post-trial compassionate givosiran treatment (Table 3). Together, the reduction in biochemical ALA/PBG precursors levels and reduced AAR suggested the disease severity was less severe after receiving givosiran treatment.

The treatment response for Pb/Gn# 2 was poor in terms of AAR, and she had medical histories and baseline characteristics worth noting. Among the 7 patients from Taiwan, she was the only one with a medical history of sepsis, and she had the highest composite AAR (25.9), and high annualized days of hemin use (45.4). She also had the highest weekly score for daily worse pain (4.89) during the 6-month placebo and through treatment (4.03 through month 12) (**Supplement Table 1**). Clinically meaningful change in SF-12 PCS score from baseline was only marginal (2 points improvement). Further, Pb/Gn# 2 had a severe SAE of sepsis and a nonserious AE of phlebitis during the clinical trial (all considered unrelated to the treatment drug). Despite the composite AAR during givosiran treatment (10) and AAR during post-trial compassionate givosiran (12) suggesting poor response to givosiran, it was lowered compared to pre-trial (17) and 6-mont placebo (25.9), and clinically extra heme arginate treatment was not always required for the attack after givosiran treatment.

## 4 Discussion

This study provides insights into the treatment efficacy of givosiran for Taiwan patients with AIP, focusing on those whose historical acute attacks can be justified with biochemical and clinical evidence before the givosiran trial. Our findings indicate that givosiran effectively reduced the level of ALA/PBG, and AAR. However, the treatment response in terms of AAR differed, this suggested that response to givosiran may be influenced by disease duration and individual baseline characteristics.

The reduction in urinary ALA/PBG levels observed in our patients during post-trial compassionate givosiran treatment compared to pre-trial level, even during acute attacks, suggested a biochemical improvement. However, it is important to note the elevations or reduction in biochemical markers alone was not enough to assess the severity of the attack. While urinary PBG/ALA levels generally elevate in acute attacks and gradually decrease during treatment, it is known for some patients who were more severely affected by frequent acute attacks, their urinary PBG/ALA levels may remain higher than the reference range even during remission. [17,18] Likewise, frequent episodes of abdominal pain do not equal having frequent acute attacks. For this reason, we emphasize that biochemical and clinical evidence is needed to confirm the occurrence of an acute attack.

Treatment response in terms of reduction in AAR was observed in our patients, however, only the patient who had a shorter disease duration (1.6 years since AIP diagnosis) and no prior prophylactic treatment had a zero AAR during givosiran treatment. This may suggest that earlier intervention with givosiran once the patients' eligibility for prophylaxis was confirmed, may lead to better clinical outcomes in terms of reducing attack frequency. This finding is consistent with a recent multicenter study which reported givosiran was more effective when given early in the disease course. [15]

Our finding of better treatment response for patient without prior prophylaxis treatment was different from the previous studies [[14], [19]]. In our study, we observed that despite all patients having a reduction in urinary ALA /PBG levels during givosiran treatment, the patient who had received weekly prophylactic heme arginate infusion until the washout period had poor treatment response in terms of AAR, and the marginal clinically meaningful improvement in SF-12 score. The study by Bonkovsky et al., 2020, reported that patients who had received hemin prophylaxis before the givosiran trial showed substantial clinical benefits when treated with givosiran, similar to those without previous hemin prophylaxis [19]. The recently published final analysis from the phase 3 study also reported no difference in the proportion of patients with an AAR that was lower than historical AAR by 3-month interval and remained lower through the end of the study between those with and without a history of hemin prophylaxis, [14] and improvement in SF-12 scores was observed regardless of prior hemin prophylaxis status. [14] The difference could be due to different study populations, or may suggest that the patients experienced a similar quality of life during givosiran treatment compared to their previous prophylactic treatment. Due to the small number of patients with AIP treated with givosiran in Taiwan, further research is necessary to confirm our preliminary finding.

The final analysis from the phase 3 clinical trial on givosiran reported an acceptable safety profile. [14,16] The most frequently reported treatment-related adverse events (AEs; ≥10 %) over the 36-month trial period were injection–site reactions, nausea, and fatigue, and AEs were mostly mild or moderate in severity. [14, 16]In our study, the AE was unremarkable and there was no AE that led to discontinuation of treatment. One patient (Pb/Gn#2) who had a medical history of sepsis, had a severe SAE of sepsis and a nonserious AE of phlebitis. These AEs were considered unrelated to the givosiran treatment. Nevertheless, concerns have been reported regarding the relatively high occurrence of adverse events associated with givosiran treatment, including fatigue (68 %), nausea (40 %), and injection-site reaction (26 %). [15] Acute pancreatitis was also reported. [15,20] For this reason, personalized adaptations of givosiran therapy based on ALA levels administered have been suggested. [15] In their study, 42 % of patients with stable low levels of ALA were followed and givosiran injection was only scheduled when ALA increased to a threshold of 10 μmol/mmolCr. [15] Our recent study supports this view and emphasizes the importance of having objective biochemical evidence for defining a requirement for givosiran treatment. [6,8]

While the ease of givosiran administration and absence of risk for iron accumulation offer some distinct advantages over hemin prophylaxis, [2,14] the drawbacks of givosiran include its significantly higher cost of care compared to hemin, [21] and potential hepatic and renal adverse effects such as hyperhomocysteinemia. [22,23] A recent long-term follow-up study of givosiran treatment reported an acceptable safety profile for patients with AIP receiving monthly givosiran therapy for up to 4 years, with one mild case of elevated homocysteine which did not require modification of givosiran dosing, [24] suggesting givosiran has acceptable safety for patients with AIP, particularly for patients with concerns about adverse events associated with prolonged or repeated use of hemin. In our study, none of the patients required dosing change or discontinuation due to changes in homocysteine level during givosiran treatment (the homocysteine level for patient Pb/Gn #1 was 48.2 μmol/L during givosiran treatment and 15.4–27.8 μmol/L after discontinuing givosiran; Patient Pb/Gn #2 was 32.7 μmol/L during givosiran treatment and 9.5–12 μmol/L after discontinuing givosiran). However, the long-term consequences of elevated homocysteine levels in patients with AIP remain unknown, and further research into the dysregulation of cysteine metabolism in AIP patients is necessary, potentially refining its use to those with the most critical disease burden and tailoring approaches to individual metabolic profiles.

Despite the consensus on the safety and effectiveness of givosiran, given potential limitations on medical resources, [21] further research is necessary to identify patients who are likely good responders to givosiran, and to investigate personalized dosing intervals. Our study reported that disease characteristics including years since AIP diagnosis, and prior use of prophylaxis regimen may influence treatment response and quality of life. Given the small number of patients who have received givosiran treatment in Taiwan, our findings must be validated with a larger sample size.

The major limitation of this study is the small patient size, which reduces the power of the study and the finding cannot be generalized to the Western population. However, due to the rarity of the disease in Taiwan as well as the fact that givosiran is not yet approved in most East Asian countries, our longitudinal biochemical data and clinical data collected before the trial offered important contributions to the management of this rare genetic disease.

## 5 Conclusions

This study focused on the treatment response of two cases of AIP patients whose pre-clinical AAR was confirmed with biochemical and clinical evidence. Our data suggests that shorter disease duration and without prior prophylactic hemin infusion responded better in terms of reduction in attack frequency. However, both patients reported only marginal improvement in SF-12 score from baseline. Further studies are necessary to clarify the association between disease characteristics, treatment history, and optimal treatment response for patients with recurrent AIP in terms of both attack frequency reduction and quality of life.

## Funding

This work was supported by National Science and Technology Council, Taiwan (No. 113-2314-B-182A-141)

## Ethics approval and consent to participate

Approval was obtained from the ethics committee of Chang Gung Memorial Hospital (IRB number: 201900074B0, 202000914B0, 201800347A4, 202202356A3, 202202356A3C101, and 202202356A3C501). The procedures used in this study adhere to the tenets of the Declaration of Helsinki.

## Consent for publication

Informed consent was obtained from all individual participants included in the ENVISION study (ClinicalTrials.gov Identifier, NCT03338816).

## Permission to reproduce material from other sources

Not applicable.

## CRediT authorship contribution statement

**Hung-Chou Kuo:** Writing – review & editing, Writing – original draft, Visualization, Validation, Supervision, Software, Resources, Project administration, Methodology, Investigation, Formal analysis, Conceptualization. **Long-Sun Ro:** Writing – review & editing, Supervision, Resources, Project administration, Formal analysis. **Chia-Ni Lin:** Writing – review & editing, Writing – original draft, Visualization, Investigation. **Chun-Che Chu:** Writing – review & editing, Validation, Investigation. **Ming-Feng Liao:** Writing – review & editing, Validation, Investigation. **Hong-Shiu Chang:** Writing – review & editing, Validation, Investigation.

## Declaration of competing interest

The authors declare that they have no known competing financial interests or personal relationships that could have appeared to influence the work reported in this paper.

Hung-Chou Kuo is a principal investigator in the ENVISION study.

## Data Availability

The data that support the findings of this study are not openly available due to reasons of sensitivity but are available from the corresponding author upon reasonable request. Data are located in controlled access data storage at the Department of Neurology, Chang Gung Memorial Hospital & Chang Gung University.

## Appendix A Supplementary data

Supplement Table 1 Demographic and disease characteristics of the study cohort grouped by treatment outcome Supplementary material
