# Supplementary material for: Baseline urinary ALA and PBG as criteria for starting pharmacologic prophylactic treatment in acute intermittent porphyria treated with givosiran
Source: Mol Genet Metab Rep. 2024 Dec 5;41:101169. doi: 10.1016/j.ymgmr.2024.101169 (PMC11667067; doi:10.1016/j.ymgmr.2024.101169)
Supplement: Supplement Table 1 — Demographic and disease characteristics of the study cohort grouped by treatment outcome [file mmc1.docx]

**Supplement Table 1.** Demographic and disease characteristics of the study cohort grouped by treatment outcome (zero or non-zero median composite AAR through givosiran treatment period (36 months)

|  | Median composite AAR through givosiran treatment period (36 months) | | | | | | |
| --- | --- | --- | --- | --- | --- | --- | --- |
|  | **Zero** | | | **Non-Zero** | | | |
| **Treatment Group During DB Period** | givosiran | givosiran | givosiran | Placebo  (Pb/Gn#1) | Placebo  (Pb/Gn# 2) | givosiran | givosiran |
| **Demographics** |  |  |  |  |  |  |  |
| Age (years) | 40-44 | 20-24 | 50-54 | 25-29 | 20-24 | 25-29 | 40-44 |
| Sex | M | F | F | F | F | F | F |
| Body Weight (kg) | 66.4 | 48.7 | 48 | 59.3 | 65 | 42.1 | 58 |
| BMI | 25 | 19.8 | 17.8 | 24.4 | 26.7 | 16.4 | 21.8 |
| Type of AHP Diagnosis | AIP | AIP | AIP | AIP | AIP | AIP | AIP |
| Years Since Diagnosis | 5.6 | 4.4 | 6.9 | 1.6 | 6.7 | 9.9 | 7.4 |
| Prior hemin prophylaxis regimen | No | Yes | Yes | No | Yes | Yes | No |
| Prior chronic symptoms when not having attacks | No | Yes | Yes | No | No | No | Yes |
| Prior chronic opioid use when not having attacks | No | No | No | No | No | No | No |
| **Composite AAR** |  |  |  |  |  |  |  |
| Historical | 20 | 8 | 24 | 4 | 14 | 22 | 8 |
| During givosiran treatment (36M) | 0 | 0 | 0 | 1.7 | 7.4 | 14.5 | 0.4 |
| **Urinary ALA (mmol/mol Cr)^+^** |  |  |  |  |  |  |  |
| Baseline | 23.34 | 13.44 | 8.81 | 17.31 | 26.72 | 9.42 | 15.71 |
| During givosiran treatment (36M) | 0.98 | 0.32 | 0.32 | 0.88 | 2.8 | 1.32 | 0.46 |
| **Urinary PBG (mmol/mol Cr)^+^** |  |  |  |  |  |  |  |
| Baseline | 50.88 | 37.11 | 38.36 | 57.61 | 72.53 | 33.57 | 38.26 |
| During givosiran treatment (36M) | 2.29 | 0.28 | 0.27 | 1.1 | 15.76 | 5.06 | 0.31 |
| **Annualized Days of Hemin Use** |  |  |  |  |  |  |  |
| During DB | **0** | 0 | 0 | 2.1 | 45.4 | 51.9 | 0 |
| During Givosiran Treatment (36M) | 0 | 0 | 0 | 0 | 10 | 64.1 | 0 |
| **Worst Pain^#^** |  |  |  |  |  |  |  |
| During DB | 1.24 | 0 | 2.26 | 1.22 | 4.89 | 1.41 | 1.35 |
| During Givosiran treatment (12M) | 0.27 | 0 | 1.37 | 0.17 | 4.03 | 1.65 | 0.49 |
| **SF-12 PCS** |  |  |  |  |  |  |  |
| Baseline | 48.18 | 51.10 | 27.99 | 49.17 | 50.61 | 46.59 | 45.40 |
| During Givosiran treatment (36M) | 57.62 | 59.87 | 28.54 | 52.04 | 52.61 | 35.96 | 61.0 |
| Difference from baseline***** | 9.44 | 8.77 | 0.55 | 2.87 | 2.00 | -10.63 | 15.60 |

Data were presented as median and (minimum, maximum) or count (percentages).

Composite AAR: composite annualized attack rate. Porphyria attacks requiring hospitalization, an urgent healthcare visit, or intravenous hemin treatment at home within 6 months before randomization.

Historical: within 6 months before randomization

**#** Average weekly score for the daily worst pain, patients recorded their daily pain by eDiary from screening through Month 12, which included the 6-month double blind (DB) period and first 6 months of the open-label extension (OLE) period.

*****Clinically meaningful difference was defined as an increase of >2 points

AAR, annualized attack rate; AIP, acute intermittent porphyria; ALA, aminolevulinic acid; BMI, body mass index; Cr, creatinine; DB, double blind period; PBG, porphobilinogen; SF-12 PCS: Physical Component Summary (PCS) of the 12-item Short-form Health Survey (SF-12)

**^+^**ALA reference range (ULN, 1.47 mmol/mol Cr); PBG reference range (ULN, 0.14 mmol/mol Cr)
